# Prokaryotic microvesicles Ortholog of eukaryotic extracellular vesicles in biomedical fields

**DOI:** 10.1186/s12964-023-01414-8

**Published:** 2024-01-30

**Authors:** Halimeh Mobarak, Farzin Javid, Maryam Taghavi Narmi, Narges Mardi, Fatemeh Sadeghsoltani, Parisa Khanicheragh, Samaneh Narimani, Mahdi Mahdipour, Emel Sokullu, Ferzane Valioglu, Reza Rahbarghazi

**Affiliations:** 1 Department of Applied Cell Sciences, Faculty of Advanced Medical Sciences, Tabriz University of Medical Sciences, Tabriz, Iran; 2 Biotechnology Research Center, Tabriz University of Medical Sciences, Tabriz, Iran; 3 Department of Clinical Biochemistry and Laboratory Medicine, School of Medicine, Tabriz University of Medical Sciences, Tabriz, Iran; 4 Student Research Committee, Tabriz University of Medical Sciences, Tabriz, Iran; 5 Stem Cell Research Center, Tabriz University of Medical Sciences, Tabriz, Iran; 6 Biophysics Department, Koç University School of Medicine, Rumeli Feneri, 34450, Sariyer, Istanbul, Turkey; 7 Technology Development Zones Management CO, Sakarya University, Sakarya, Turkey

**Keywords:** Exosomes, Bacterial microvesicles, Prokaryotic cells, Eukaryotic cells, Biological properties, Biomedical applications

## Abstract

Every single cell can communicate with other cells in a paracrine manner via the production of nano-sized extracellular vesicles. This phenomenon is conserved between prokaryotic and eukaryotic cells. In eukaryotic cells, exosomes (Exos) are the main inter-cellular bioshuttles with the potential to carry different signaling molecules. Likewise, bacteria can produce and release Exo-like particles, namely microvesicles (MVs) into the extracellular matrix. Bacterial MVs function with diverse biological properties and are at the center of attention due to their inherent therapeutic properties. Here, in this review article, the comparable biological properties between the eukaryotic Exos and bacterial MVs were highlighted in terms of biomedical application.

Video Abstract

**Supplementary Information:**

The online version contains supplementary material available at 10.1186/s12964-023-01414-8.

## Introduction

In the course of the evolution process, numerous biological mechanisms have been created to help prokaryotes and eukaryotes survive by using inter- and intra-species interaction [1]. During the last decades, the critical role of extracellular vesicles (EVs) has been proved in reciprocal cell–to–cell communication in a paracrine manner [2–4]. From the ultrastructural aspect, the term EVs encompasses heterogeneous bilayered nano-sized vesicles with the potential to carry numerous signaling molecules to promote synchronous multicellular dynamic growth and function [5, 6]. The production and release of EVs are thought to be a conserved biological phenomenon almost in all types of eukaryotic and prokaryotic (Gram-negative, and Gram-positive bacteria) cells [1, 7, 8].

**Fig. 1:**
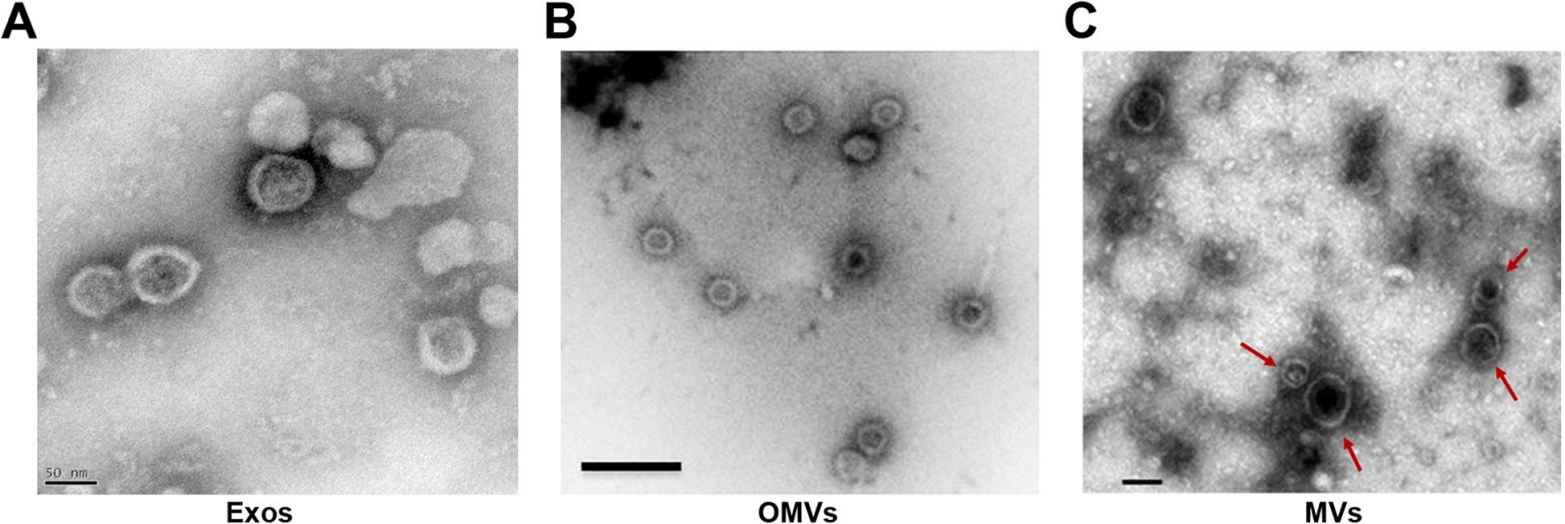
Similarities between eukaryotic and prokaryotic EVs (**A**-**C**). Ultrastructural analysis of human bone marrow mesenchymal stem cell Exos (**A**). Images revealed cup-shaped and nearly round morphology in isolated Exos [30]. (Copyright 2021, Frontiers in Cell and Developmental Biology). TEM images of *Capnocytophaga ochracea* OMVs at stationary phase after exposure to pH value of 5.1 (**B**: Scale bar: 200 nm) [31]. (Copyright 2021, Frontiers in Microbiology). MVs (red arrows) were isolated by ultracentrifugation from *Staphylococcus aureus* cultured in Tryptic Soy Broth medium (**C**: Scale bar: 100 nm) [32]. (Copyright 2018, Nature Communications)

Almost all types of unicellular structures such as archaea, bacteria, viruses, fungi, and parasites can produce MVs and release them to the microenvironment [9–22]. Membrane vesicles (MVs) are heterogenic in terms of morphology, size, and cargo type similar to eukaryotic EVs (Fig. 1, Table 1) [7, 23–25]. MVs structure in pathogenic and non-pathogenic Gram-negative bacteria, known also as outer membrane vesicles (OMVs), are nano-sized and originate from the cells’ outer membrane (OM). Whereas, Gram-positive bacteria-derived MVs are released from the single cytoplasmic cell membrane surrounded by a peptidoglycan-rich cell wall [26]. It has been postulated that microorganism EVs contain virulent factors and pathogenic compounds that help in the progression of infection in the host cells. Besides, microorganism EVs are involved in the regulation of the immune system, and the neutralization of antibiotics and bacteriophages [27, 28]. To date, there is a gap in terms of microorganism EV impacts on multicellular systems and how and whether these EVs can regulate the function of eukaryotic cells under physiological and pathological conditions. The most of previously conducted studies have been limited to the evaluation of eukaryotic EVs, especially humans and other species. It is thought that focusing on microorganism EVs and their interaction with eukaryotic systems can give us an insightful vision of biological phenomena that occur following the activation of single-celled organisms inside the metazoan niche [7, 8, 29]. The existence of paracrine interaction between the cells in multicellular (metazoan) and unicellular creatures via EVs highlights the fact that the vesicular transmission of signaling molecules is a conserved biological phenomenon. It is recommended biologists evaluate ultrastructural and cytochemical features and biogenesis signaling pathways of eukaryotic and prokaryotic EVs. Here, in this review article, we tried to scrutinize the distinct properties of prokaryotic EVs and their physiological roles in comparison with eukaryotic EV counterparts.

**Table 1:** Comparison between Eukaryotes, Prokaryotes, and Archaea EVs

Domains of life		Origin	Diameter (nm)	Terms
**Eukaryotes**	Mammalian cells	Apoptosis process: with origin cell membrane and components	1000–5000	Apoptotic bodies (ABs)
		The outward budding of the cell membrane	100–1000	Microvesicles, Extracellular membrane vesicles, Microparticles, Ectosomes, Exovesicles
		Endosomal origin: early and late endosome for intraluminal vesicles (ILVs) formation then Multivesicular Bodies (MVB) fusion with the cell membrane	30–150	Exosomes (Exos)
**Prokaryotes**	Gram-negative bacteria	Outer membrane	10–500	Outer membrane vesicles (OMVs)
	Gram-positive bacteria	Cytoplasmic membrane	20–400	Membrane vesicles (MVs)
**Archaea**	Archaea	Cytoplasmic membrane	50–230	Membrane vesicles (MVs)

## Eukaryotic EVs

**Fig. 2:**
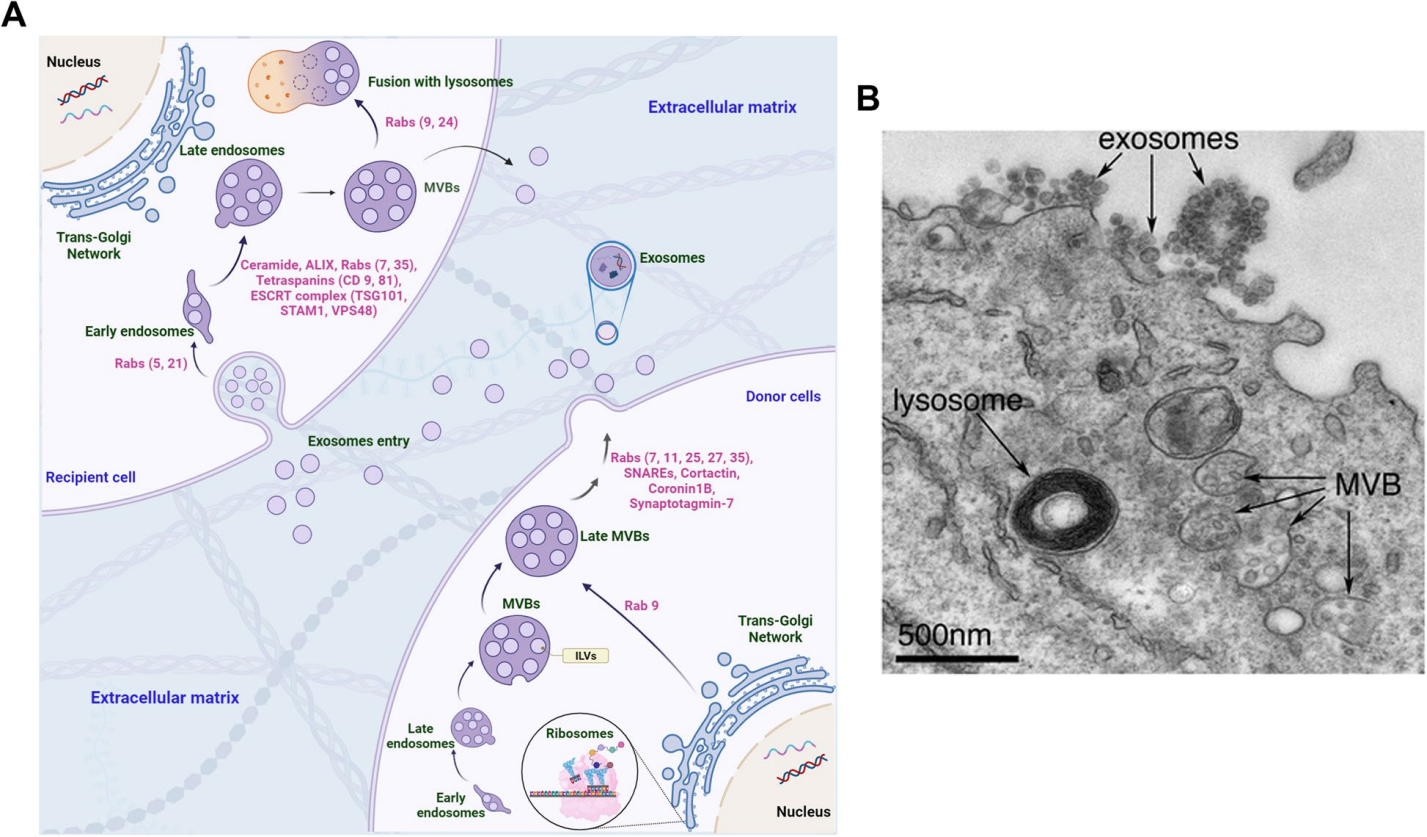
Exosome (Exo) biogenesis pathway (**A**). Exos are generated and released using the endosomal system. In the recipient cells, internalized Exos are sequestrated inside the early endosomes followed by maturation into the mature endosomes. Inside the mature endosomes, numerous intraluminal vesicles (ILVs) are generated via the invagination of the endosomal membrane. In this step, several signaling molecules are sequestered into the ILV lumen. After that, late endosomes can mature into MVBs. In the following steps, MVBs can fuse with lysosomes for content degradation or make close connections with cell membranes for the release of ILVs into the extracellular matrix where they hereafter are known as Exos. Ultrastructural images of B lymphocyte with expelled Exos at the plasma membrane. MVBs can be directed toward lysosomal degradation or release their content into the extracellular matrix. Released ILVs (black arrows) are named Exos out of the parent cells. Reprinted adapted from [35]. (Copyright 2016, BMC Biology)

In eukaryotes, three types of EVs have been identified based on biogenesis, content, and size of vesicles. Among EVs, apoptotic bodies exhibit an average diameter of 1000 to 5000 nm and are produced during the activation of the apoptotic process via the disassembly of the cell membrane. Microvesicles, also known as microparticles, ectosomes, and exovesicles, range between 100 to 1000 nm and are generated by the evagination of cell membranes under physiological and pathological conditions in response to diverse stimuli. The last and smallest EV type is named exosomes (Exos) with an average diameter between 30 and 150 nm. Exos are produced by the activity of the endosomal system and released to the extracellular matrix (ECM) after the fusion of endosomes with the cell membrane (Fig. 2). Upon the invagination of the cell membrane, early endosomes are generated with numerous Exos entering the host cells. Molecular investigations have revealed that early endosomes can be directed toward the Golgi apparatus and/or lysosomal degradation. In an alternative pathway, early endosomes mature into late endosomes where numerous intraluminal vesicles (ILVs) are generated via the invagination of the endosomal membrane. The phenomenon is continued by the maturation of late endosomes toward multivesicular bodies (MVBs) where these vesicles can commit lysosomal degradation or fuse with the cell membrane to release luminal cargo of ILVs into ECM, hereafter called Exos. To date, the isolation and purification of EV types is one of the most challenging issues in biomedical fields. Practically, the isolation and purification of the EV subpopulation are not possible because of overlapping density, diameter size, cargo type, and even lack of definite markers [3, 33, 34].

Molecular investigations have revealed the existence of specific proteome [growth factors, cytokines, proteins, and enzymes], transcriptome [mRNAs, microRNAs], and lipid contents in the lumen of eukaryotic EVs [36, 37]. Besides their roles in mutual cell-to-cell interaction under different conditions, EVs are thought as valid diagnostic tools for the detection and monitoring of certain pathologies. Using engineering modalities, EVs can be used for the on-target delivery of therapeutics such as drugs, genes, and certain immunogens with promising therapeutic outcomes [38, 39]. It was suggested that EVs can be actively involved in the progression of distinct pathological conditions such as anaplastic changes, neurodegenerative, infectious cardiovascular diseases, and senile changes [7, 40, 41].

## Bacterial EVs

**Fig. 3:**
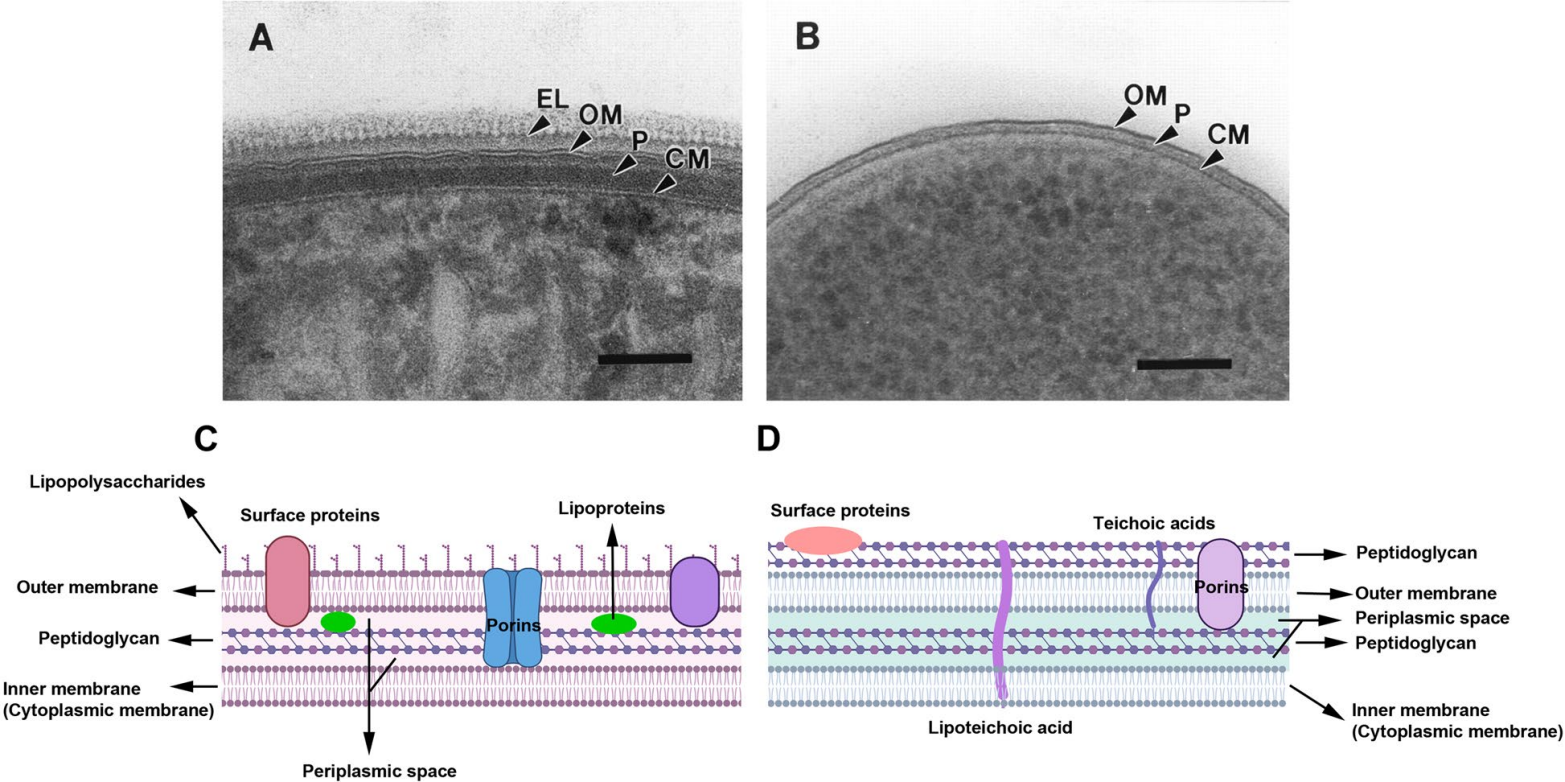
Ultrastructural TEM images of cryo-sectioned Gram-negative cell envelopes. **A**: *Cyanobacterium Phormidium uncinatum* and **B**: *Escherichia coli*. *Cyanobacterium Phormidium uncinatum* has a combination of Gram-positive and –Negative structures. Note the thick peptidoglycan layer and outer membrane. The external layer (EL) consists of S- layer and oscillin fibrils with serrated surface morphologies (Scale bar: 100 nm). Reprinted adapted from [50]. (Copyright 2000, Journal of Bacteriology) [Cytoplasmic membrane: CM; outer membrane: OM; and Peptidoglycan: P]. Typical cell wall structure in Gram-negative (**C**) and Gram-positive bacteria (**D**)

As abovementioned, bacteria can also secrete nano-sized EVs or MVs which are heterogeneous based on size, density, amount, and cargo component within the same species. It was suggested that the growth phase, niche condition, and several external stimuli can affect the quality of MV production in bacteria [28, 42, 43]. Noteworthy, the heterogeneity is related to engaging different biogenesis pathways, membrane structure of the parent bacteria, growth conditions, and genetic traits [44]. Gram-negative bacteria possess an OM harboring lipopolysaccharide (LPS), and a thin periplasmic peptidoglycan layer in the periplasmic space (Fig. 3). The peptidoglycan composites, known also mureins, are located between the outer and inner membranes [45]. These bacteria also contain a hard biopolymer peptidoglycan in the cell wall which is involved in the production of EVs. Along with these comments, Gram-negative bacteria OMVs have a large amount of LPS, proteins (cytoplasmic, periplasmic, and membrane-bound), outer membrane lipids, virulence factors, immunomodulatory factors, ribonucleic acids, toxins, and other pathogen-associated molecular patterns (PAMPs) (Fig. 3 and Table 2) [1, 24, 28, 46–49]. It is speculated that the introduction of MV PAMP contents to host pattern recognition receptors (PRR) in immune/non-immune cells results in immune tolerance, pathological conditions, and protective immunity [44]. Some of the PRRs located in the intestinal epithelial cells contain the cytoplasmic nucleotide-binding oligomerization domain (NOD) and transmembrane Toll-like receptors (TLRs). Either TLR or NOD-like receptor (NLR) families are stimulated in response to bacterial EVs with crucial roles in OMV/MV-mediated pathologies [26].

**Table 2:** The function of MVs in Gram-negative/positive bacteria and archaea

Functions	Domain	Species	References
Delivery of toxic factors to eukaryotes/prokaryotes	Gram-negative bacteria	*Pseudomonas aeruginosa, Bordetella pertussis, Escherichia coli, Aggregatibacter actinomycetemcomitansm, Vibrio cholera, Pseudomonas fragi, Kingella kingae, Salmonella enterica pullorum, Pantoea agglomerans, Proteus vulgaris, Morganella morganii, Phyllobacterium trifolii*	[51–58]
Exchange of proteins (e.g., OM proteins (OmpA, OmpC, and OmpF), periplasmic proteins (alkaline phosphatase and AcrA), Hemin binding protein C and virulence factors (adhesins, invasins, and other enzymes) between cells	Gram-negative bacteria	*Neisseria meningitides, Escherichia coli,* *Gemmata obscuriglobus, Bartonella henselae*	[59–62]
Exchange of lipids (e.g., glycerophospholipids, phosphatidylethanolamine, phosphatidyl-glycerol, and cardiolipin) between cells	Gram-negative bacteria	*Escherichia coli, Pseudomonas syringae, Myxococcus xanthus*	[63–65]
	Gram-positive bacteria	*Bacillus anthracis,* *Staphylococcus aureus*	[66, 67]
Biofilm production	Gram-negative bacteria	*Pseudomonas aeruginosa, Helicobacter pylori*	[68–70]
Response to stress	Gram-negative bacteria	*Escherichia coli* *Salmonella typhimurium, Pseudomonas aeruginosa*	[71–73]
Transfer of genetic materials (DNA/RNA)	Gram-negative bacteria	*Pseudomonas aeruginosa*, *Escherichia coli, Neisseria gonorrhoeae, Borrelia burgdorferi, Haemophilus influenza, Moraxella osloensis, Salmonella typhimurium, Serratia marcescens,* Shigella spp. (*Shigella dysenteriae,* and *Shigella flexneri*), *Shewanella vesiculosa*, *Porphyromonas gingivalis, Yersinia pestis,* and *Vibrio cholerae*	[74–80]
	Gram-positive bacteria	Ruminococcus spp. Mycobacteriaceae	[81, 82]
	Archaea	Thermococcales (*Thermococcus kodakarensis*, and *Nautilia lithotrophica*)	[83, 84]
Promote antibiotics resistance	Gram-negative bacteria	*Porphyromonas gingivalis, Pseudomonas aeruginosa,* *Pseudomonas putida*, *Escherichia coli*	[85–88]

The existence of OMVs was first indicated in the Gram-negative bacterium (*Escherichia coli*) in 1966. After that, OMVs were detected in patients’ cerebrospinal fluid with meningococcal disease. Later, the Gram-negative pathogens OMVs originated from specific species such as *Neisseria meningitides, Helicobacter pylori*, and *Haemophilus influenza* were confirmed [26]. Microorganism EVs produced by other prokaryotes such as Gram-positive bacteria, and other archaea are called MVs [89].

In Gram-positive bacteria, a thick layer of peptidoglycan surrounds the cell membrane with no outer membrane [90]. Some phyla such as Mycobacteria, and Actinobacteria, are completely different from Gram-positives and Gram-negatives. For instance, ultrastructural analyses have revealed that three main macromolecules – peptidoglycan, arabinogalactan (a highly branched polysaccharide), and mycolic acids (a specific lipid component in the cell wall), made the core structure of the mycobacterial cell wall. Because of the high density of lipids in their cell wall, they are also mentioned as acid-fast bacteria [91]. Gram-positive bacteria MVs originate from cell membranes with cytoplasm- and membrane-associated proteins and factors [89]. The existence of specific protein coating, namely the S layer, in Gram-positive and negative bacteria leads to physical interaction between the bacteria and the neighboring niche. Notably, it is suggested that the bacterial S layer can be eliminated after a prolonged culture period used for MV isolation [92].

**Fig. 4:**
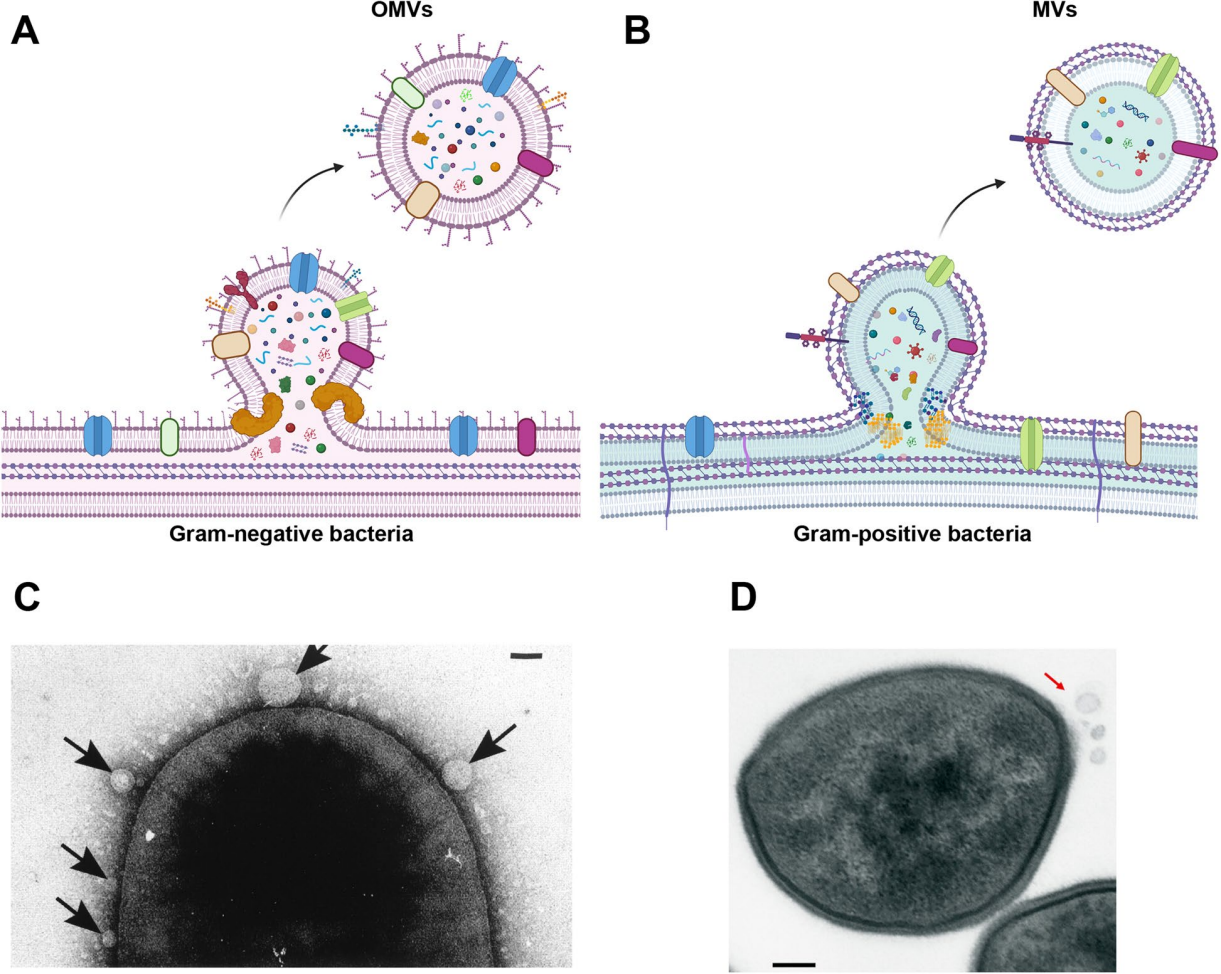
Steps for the generation of OMVs and MVs in Gram-negative (**A**) and Gram-positive (**B**) bacteria, respectively. TEM image of production of MVs (black arrows) by Gram-negative bacterium *Serratia marcescens* (**C**). Samples were stained with 2% uranyl acetate solution and imaged at 60 kV (Scale bar: 100 nm). Reprinted adapted from [52]. (Copyright 1998, Journal of Bacteriology). Ultrastructural imaging revealed the formation of MVs (red arrows) on the cell wall of *Staphylococcus aureus* cultured in the Tryptic Soy Broth medium (Scale bar: 100 nm) [32]. (Copyright 2018, Nature Communications)

As a common belief, most archaea species can constitutively produce MVs with an average size of 50–250 nm (Fig. 4). This phenomenon is initiated via the bulging of cell membranes using electron tomography and micrographs in *Sulfolobus* and *Thermococcales* species [83, 93]. It should not be neglected that MVs are not confined to extra-organism niches. These natural nanoparticles can also participate in intra-bacterial metabolism. For instance, numerous MVs have been identified in the periplasm space of Ignicoccus species. It is thought that these MVs play a key role in the transfer of several signaling molecules between the inner and OM. Of note, ultrastructural analyses have revealed the lack of an S layer in *Ignicoccus* species [94].

Like eukaryotic EVs, the production of MVs is constituent in different conditions. For example, the release of OMVs has been documented after in vitro microbiological cultures (biofilms, etc.) or in vivo expansion in biofluids (cerebrospinal fluid, and blood) and solid tissue samples (gastric tissue) [8, 95]. To resist the insulting conditions, most bacteria exhibit communities and attach to beneath a slim layer of biofilm composed of polysaccharides, proteins, and genetic elements. Emerging data have revealed the involvement of OMVs in the formation of biofilms [89]. *Pseudomonas aeruginosa* can prolong pulmonary infection after the production of biofilm [68]. Data confirmed that OMVs can promote the formation of ECM by the transport of adherent substances and nutrients. In support of this notion, OMV-producing bacteria have robust biofilm generation capacity compared to OMV-free stains [85]. In an experiment, it was found that the relationship between distinct properties such as extracellular ATP, MV release and biofilm, and bacterial viability was investigated. Based on the obtained data, the existence of such conditions potentiates the *Shewanella vesiculosa* M7T strain to resist harsh niches such as Antarctica [49]. As described for eukaryotic EVs, MV cargo can differ based on the parent organism, strain, and mechanism of production [49]. Therefore, it is logical to postulate that these parameters can affect MV cargo such as the content of bacterial pro-inflammatory factors [LPS, OM proteins (OMPs), periplasmic compounds, polysaccharides, nucleic acids, and phospholipids] [47, 96, 97]. Based on previous data, phosphoglycerolipids (PLs), the most abundant lipids, exist at high levels inside the OMVs but not MVs [98]. For this purpose, Gram-negative bacteria OMVs have been used for the fabrication of vaccines [99]. The transfer of LPS by OMVs to the immune cells leads to the production of pro-inflammatory cytokines without the emergence of severe pathological responses [100]. Besides, MVs can be donated and received for the modulation of bacterial activity. Of course, the mutual transfer of MVs is not the sole pathway for the interchange of varied substances between bacteria. Recent data have indicated the formation of tubular structures namely nanotubes between bacteria from the same and different species for reciprocal interchange of biomolecules [101]. It seems that the formation of nanotubes is done in both Gram-negative and Gram-positive bacteria. For example, genetic materials such as DNA, toxin-related tRNAase, and plasmids can be transferred via nanotubes in *Bacillus subtilis* which lacks an OM. Other species such as *Staphylococcus aureus* and *Escherichia coli* have the potential to maintain inter-bacterial interchange via nanotubes [101, 102].

## Biogenesis of MVs in bacteria

It has been postulated that several molecular mechanisms are involved in the formation of bacterial EVs with different subtypes, specific cargoes, and biological functions [44]. In short, Gram-negative bacteria usually use two basic pathways for EV formation. In the first mechanism, bacterial OM is an envelope for the released OMVs while the second mechanism consists of explosive bacterial lysis leading to the generation of outer-inner membrane vesicles (OIMVs) and explosive outer-membrane vesicles (EOMVs) [44, 98]. Gram-positive bacteria, the cytoplasmic membrane vesicles (CMVs) are produced via endolysin-triggered bubbling cell death [98]. The membrane blebbing pathway causes the formation of OMVs via the disruption of cross-junctions between the OM and the underlying peptidoglycan cell wall [44].

Emerging data have indicated that the activity of certain enzymes in Gram-positive bacteria leads to the weakening of cell wall peptidoglycan structure and further release of MVs [103]. The phenomenon of *Staphylococcus aureus* MVs releasing was first approved using ultrastructural electron microscopy analyses. Data indicated the disruption of the peptidoglycan layer that potentiates cytoplasmic membrane protrusion via cell wall, and MVs release [90]. To be specific, local accumulation of lipids loosens the physical connection of OM with the beneath peptidoglycan. Along with these changes, the concentration of peptidoglycan components at the site of MV formation increases the sequestration of protein inside the MVs. The activity of integral membrane proteins and certain OM molecules leads to lipid remodeling and vesicle formation [74, 104–106]. The accumulation of phenol-soluble modulins (PSMs) in the cytoplasmic membrane results in MV formation by increasing membrane fluidity [26].

In Gram-negative bacteria, the OM-peptidoglycan connection is loosened in specific sites without proper attachment. Various misfolded proteins are also recruited and accumulated as nanodomains in the sites with less content of peptidoglycans. With the progression of these changes, molecular changes in LPS facilitate fluidity and bulging in OM, resulting in the release of OMVs based on certain phospholipid types [107]. Gram-positive bacteria MVs directly protrude from the cell membrane, thus containing a large number of cytoplasmic compounds [98, 108]. It is possible these harbor several toxins [109], peptides [110], and genomic elements [74, 110, 111]. Most previously conducted experiments on MVs have been dine in certain species like *Staphylococcus aureus* [90], and *Bacillus* spp. [109, 112], *Streptococcus* spp. [113–115], *Listeria monocytogenes* [116, 117], and *Clostridium* spp. [111, 118]. Preliminary data on Gram-negative bacteria OMVs have been obtained from strains of *Escherichia coli*. It should not be forgotten that released OMVs carry some identical factors related to host bacteria. For instance, researchers claimed that the releasing MVs in *Escherichia coli* spp. encompass lipids and proteins from the mutant stains such as 12,408 [119], W3110 [120], and JC411 [121]. These features indicate that the content of release MVs can be used for immunophenotyping of parent bacteria. Avila-Calderón and co-workers highlighted the importance of lipoproteins, LPS, and peptidoglycan in OMVs biogenesis [122]. As abovementioned, they also claimed that weak lipoprotein linkages in the OM layer can increase the possibility of bulging and vesicle formation. Besides, free unbound peptidoglycan residues and the negative charge of LPS are also helpful in vesicle formation. It has been shown that specific conditions can affect the MV capacity in bacteria. For example, the phenomenon of bleb formation or bulging is increased on the external surface of *Escherichia coli* after being exposed to antibiotics such as polymyxin B [123]. Likewise, carbapenem antibiotic, imipenem, can promote high protein-content OMVs in *Acinetobacter baumannii* [124]. These data indicate a close relationship between the insulting conditions and the formation of MVs. It seems that the formation of MVs occurs in response to insulting conditions to increase bacterial resistance and circumvent the harmful factors besides their original paracrine activity in bacteria-to-bacteria communication. In support of this notion, data have indicated that the reduction and loss of antibiotics can diminish MV-related blebs in OM [122, 123]. Of course, it should not be neglected that the existence of antibiotic can also weaken the stability of OM and inner structures of bacterial membranes which per se increase MV formation. Due to existence of biomarkers inside the MVs, these nano-sized particles can be used for the diagnosis and follow-up of pathological conditions inside the body. MVs of various pulmonary tract pathogens transport specific cargoes containing nucleic acids, proteins, lipoproteins, fatty acids, glycolipids, and relevant virulence factors such as *Pseudomonas aeruginosa* CFTR inhibitory factor, *Streptococcus pneumoniae* pneumolysin, and *Legionella pneumophila* macrophage infectivity potentiator [108, 115, 125, 126]. It is possible that some factors are release by MVs and do not exist in ECM fluid. For example, macrophage infectivity potentiator and flagellin are only exist inside the bacterial MVs and cannot be detected in ECM. In an experiment, it was suggested that in Gram-positive bacteria such as *Bacillus subtilis* and *Streptococcus pyogenes*, MVs biogenesis depends on prophage-encoded endolysin cell death with simultaneous cell wall peptidoglycan disruption. This biological process involved in Gram-negative bacteria OMV production such as *Pseudomonas aeruginosa,* is prophage-mediated explosive cell lysis. In both mechanisms, the integrity of the membrane is blunted and the production of MVs/OMVs associated with defective prophage activation through genotoxic stress [26, 127].

Certain envelopes and environmental stresses including OM curvature-inducing structures and misfolded protein accumulation, exposure to antibiotics, and undesirable bacterial growth under improper pH values and/or temperatures can affect OMV biogenesis. A bacterial membrane integrity regulator, namely the transmembrane Tol-Pal protein system, functions as a factor in the regulation of OMV biogenesis in pathogenic and non-pathogenic bacteria such as *Escherichia coli*, *Shigella boydii,* and *Helicobacter pylori*. These studies highlighted the modifications in cell membranes such as OM blebbing and its relationship with OMV biogenesis [26, 72].

## Interactions of MVs with eukaryotes and prokaryotes

### Interactions with eukaryotic hosts

The presence of compounds such as toxins, adhesion molecules, siderophores, immune evasion proteins, and antibiotic resistance proteins in MV lumen highlights their roles in bacterial virulence [1, 128]. For example, *Listeria monocytogenes* MVs pore-forming toxin namely listeriolysin O. This toxin helps the bacterium to circumvent the host cell vacuoles [128]. The existence of various compounds inside the *Staphylococcus aureus* MVs such as lipase, protein A and SbI, staphopain A, etc. contribute to ECM degradation and bacterial invasion [1, 90, 129–132]. Pneumolysin is a pore-forming toxin inside *Streptococcus pneumoniae* MVs and is released from the infected eukaryotic cells. These features indicate the critical role of this factor in the infectivity of this bacterium [133]. Likewise, *Acinetobacter baumannii* can transfer virulence factor, Omp33–36 porin by OMVs to human immune cells such as macrophages. Data indicated that the uptake of these factors can result in apoptotic changes via the activation of Caspases and excessive autophagy response [134]. *Acinetobacter baumannii* OMVs contain other protein factors with the potential to alter other eukaryotic cell behavior such as oxidative stress in macrophages [135]. The higher levels of proteins and toxins inside the bacterial MVs lead to distance function and efficient immunomodulatory properties. Due to the existence of various delivery routes, bacterial MVs can easily enter the eukaryotic cells, resulting in the cell membrane integrity loss, and rupture of cells. Compared to free soluble factors, MVs can efficiently introduce large levels of bacterial toxins into the eukaryotic cytosol [1]. Four different mechanisms can be involved in Gram-positive bacteria MVs uptake by eukaryotic cells as follows; Direct fusion with the cell membrane, Dynamin-related endocytosis, Caveolin-mediated endocytosis, and Clathrin-related endocytosis [1, 51, 132, 136, 137]. Besides, the close interaction of bacterial MVs with cholesterol-rich domains has been also indicated. For instance, *Staphylococcus aureus* toxin A can enter the human HEp-2 laryngeal cancer cells via MVs following the application of the cholesterol-destroying agent methyl-β-cyclodextrin [136]. Wang and co-workers claimed that the uptake of *Staphylococcus aureus* MVs by THP-1 macrophages is done in a dynamin-dependent endocytosis manner in which the incubation of cells with dynasore inhibitor blunt these effects [132]. Of course, it should not be forgotten that the route of MV entry depends on the size and type of target cells [1, 138].

**Fig. 5:**
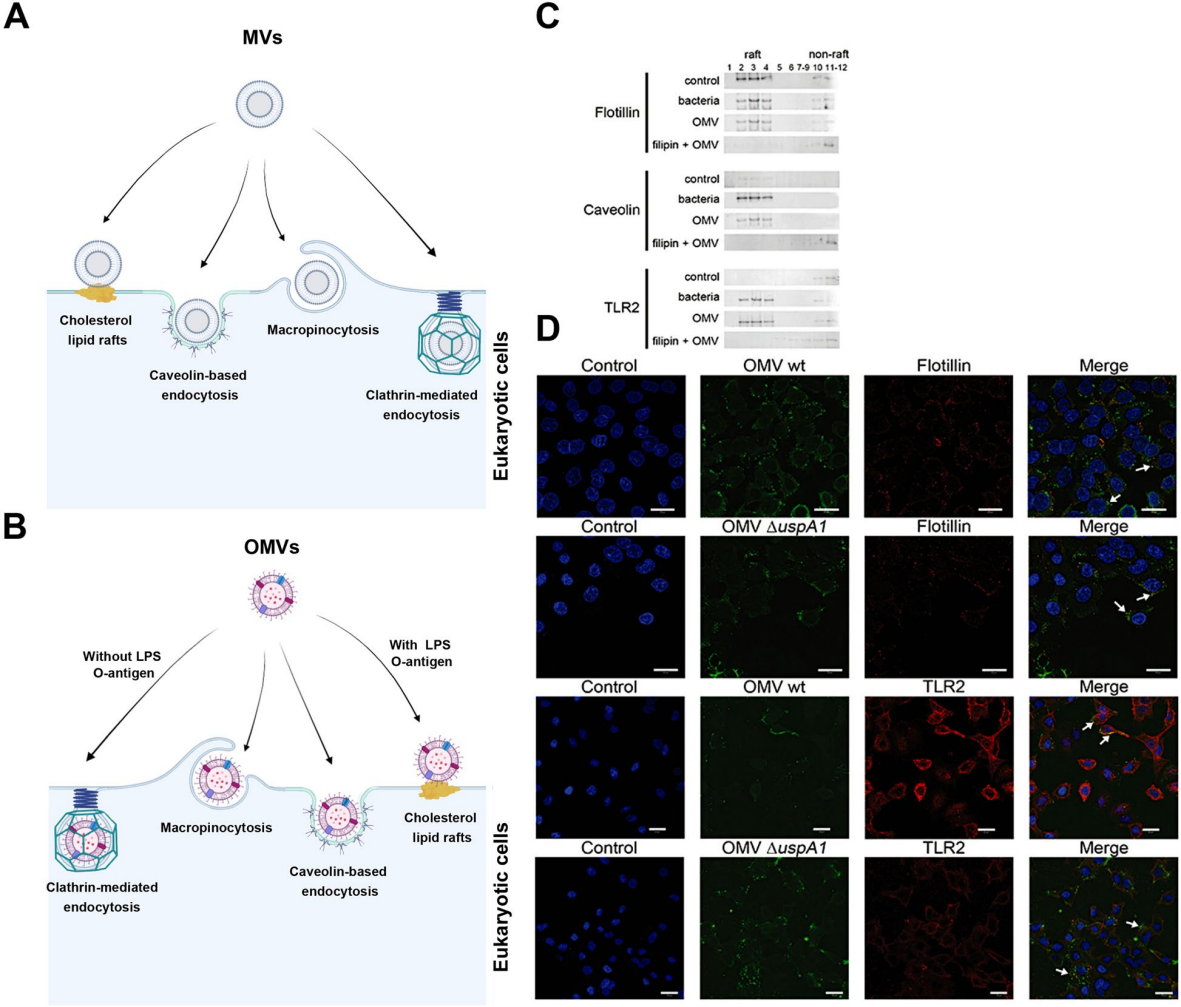
Several mechanisms of bacterial MVs internalization into the eukaryotic cells (**A**-**B**). The attachment of OMVs with eukaryotic cells led to the compartmentalization of TLR2 into the raft. Western blotting indicated that treatment of A549 cells with filipin reduced the levels of Flotillin and Caveolin, as raft fraction. Along with these changes, TLR2 clustering also diminished lipid rafts (**C**). A549 cell lysates were obtained from non-treated control, formaldehyde-treated *Moraxella catarrhalis* (named bacteria), *Moraxella catarrhalis* OMVs, and filipin-treated cells + OMVs and electrophoresed in discontinuous sucrose gradients. Immunofluorescence staining of A549 cells for monitoring receptor clustering after 1-hour treatment with OMVs from wild type and/or UspA1-deficient (OMV ΔuspA1) *Moraxella catarrhalis* (50 μg/ml) (**D**). Co-localization of OMVs occurs with flotillin and TLR2. This reaction is not associated with the activity of surface OMVs UspA1. Blue nuclei were stained with DAPI (Scale bar: 20 μm) [144]. **Abbreviations**: ubiquitous surface proteins A1 (UspA1). (Copyright 2010, Cellular Microbiology)

One of the most interesting properties of OMVs is associated with tumoricidal effects [23, 24, 139, 140]. In a study conducted by Aly and co-workers, they monitored the anticancer effects of *Salmonella typhimurium* strain ATCC14028 OMVs on human different carcinoma cell lines including colorectal, hepatocellular carcinoma, and breast cancer) in in vitro conditions and mouse model of breast adenocarcinoma (Ehrlich solid carcinoma). The incubation of tumor cells with *Salmonella typhimurium* OMVs led to the reduction of tumor mass, cancer cell dynamic growth, and induction of apoptosis (Caspase-3↑ and Bax↑), autophagy (Beclin-1↑), and activation of CD49b^+^ pan-NK cells. Along with these changes, the expression of genes associated with proliferation (Ki-67↓), and angiogenesis (VEGF↓) was also inhibited [140]. These data support the oncostatic effects of bacterial OMVs as a safe alternative therapeutic option along with conventional medications. Whether and how OMVs can exert anti-tumor effects has been not completely addressed. In an interesting experiment, single-dose administration of attenuated *Salmonella typhimurium* OMVs (0.11 mg/kg) and photothermal therapy in mice bearing colon (CT26 cells) and breast cancer cells (4 T1 cells) led to blackening of tumor mass in the early hours after treatment. One reason for these effects would be that OMVs cause massive RBC extravasation in the tumor vascular system, resulting in the loss of blood support and effective therapeutic outcomes [24]. Despite therapeutic outcomes, it is postulated that OMVs should be used in in vivo conditions with some caution. For instance, underestimation of OMV doses can lead to immunotoxicity. To this end, synthetic bacterial vesicles (SyBVs) have been developed by using chemical engineering of lysozyme, detergents at high pH values in in vitro conditions without any toxic effects on immune cells. To be specific, the levels of DNA, RNA, cytosolic factors, and other biological contaminants were trivial in SyBVs. To yield proper tumoricidal effects, co-administration of SyBVs and tumor cell EVs is suggested [47]. The entry and on-target uptake of MVs should not be neglected. Data have indicated that hydrophilicity and net electrostatic charge are key elements in the attachment of MVs to eukaryotic cells and other bacterial strains [97]. Interestingly, *Pseudomonas aeruginosa* OMVs can attach and fuse with the OM of Gram-negative bacteria (*Escherichia coli*, *Salmonella typhi*, and other *Salmonella enterica* spp.), leading to the transfer of OMV cargo into periplasm space. While Gram-positive bacteria (*Staphylococcus aureus*, *Bacillus subtilis*, *Listeria monocytogenes*, and *Enterococcus hirae*) cannot adsorb the OMVs. The reason would be that the net charge of *Pseudomonas aeruginosa* OMVs is negative which blunts their physical interaction with the surface of Gram-negative bacteria. At physiological pH values, the surface charge of Gram-negative bacteria is highly increased due to LPS, and numerous free carboxyl and amino terminus. Due to the suitable hydrophilic surfaces of *Bacillus subtilis*, OMVs can easily attach to bacterial surfaces and enter as compared to other gram-positive bacteria [45, 97, 141]. Generally, many pathways are involved in the uptake of OMVs/MVs into target cells, including direct fusion to the plasma membrane, dynamin-, caveolin-, and clathrin-mediated endocytosis, phagocytosis, lipid-raft-dependent and lipid-raft-independent endocytosis, and macropinocytosis (Fig. 5). It seems that MVs use several routes to enter the target cell especially depending on size heterogeneity. In clathrin-based endocytosis, the OMVs with a maximum size of 120 nm enter the host cells via cell surface ligands while the particles with an average size of around 1 μm cross the cell membrane via macropinocytosis [142]. Using fluorochrome-labeled bacterial EVs with several inhibitors, different internalization mechanisms have been identified [44, 143]. Notably, the composition of OMVs pre-determines the fate of these nanoparticles in the host cells. For instance, the existence of LPS and O-antigens at the periphery of OMVs activates lipid raft-dependent entry into the host eukaryotic cells [144]. In vitro analyses indicated that *Moraxella catarrhalis* OMVs harbor ubiquitous surface proteins A1/A2 and Moraxella IgD-binding factor which are involved in the activation of B cells. The exposure of human pulmonary epithelial cells (A549 cell line) with *Moraxella catarrhalis* OMVs led to close interaction with lipid raft domains and internalization after the activation of TLR2 (Fig. 5) [144]. It was suggested that treatment of A549 cells with filipin promoted the disruption of cholesterol-containing lipid raft structures and prevented compartmentalization of TLR2 into the raft structure [144]. In the absence of O-antigens, OMVs can be internalized using clathrin-mediated endocytosis [145]. In an experiment conducted by O’Donoghue and co-workers, they claimed that the inhibition of raft-mediated endocytosis using methyl-β-cyclodextrin and/or filipin reduces the *Escherichia coli* serotype O157:H7 strain Sakai 813 OMVs in HeLa cells [145]. To be specific, OMVs are internalized into the target cells using macropinocytosis, and raft- and clathrin-dependent endocytosis. However, the lipid raft is the main entry route for the bacterial OMVs in eukaryotic cells. Therefore, one could hypothesize that the dynamic growth of bacteria, environmental stress, and culture medium composition can affect the OMV composites and thus the entry route [145].

In gram-positive bacteria, three main mechanisms dynamin-, and clathrin-dependent endocytosis, and membrane fusion are possible routes for the entry of MVs into the host eukaryotic cells [1]. The inhibition of dynamin with dynasore, and dynamin-dependent endocytosis, can abolish the entry of *Staphylococcus aureus* MVs into the monocyte-macrophage lineage [132]. Likewise, the inhibition of lipid raft cholesterol using MβCD can reduce the entry rate of *Staphylococcus aureus* MVs into HepG2 cells [136]. In terms of bacterial MVs with other microbial strains, the existence of certain cell-wall attacking enzymes, especially in OMVs can facilitate the entry into the cytosol of host bacteria [103].

**Fig. 6:**
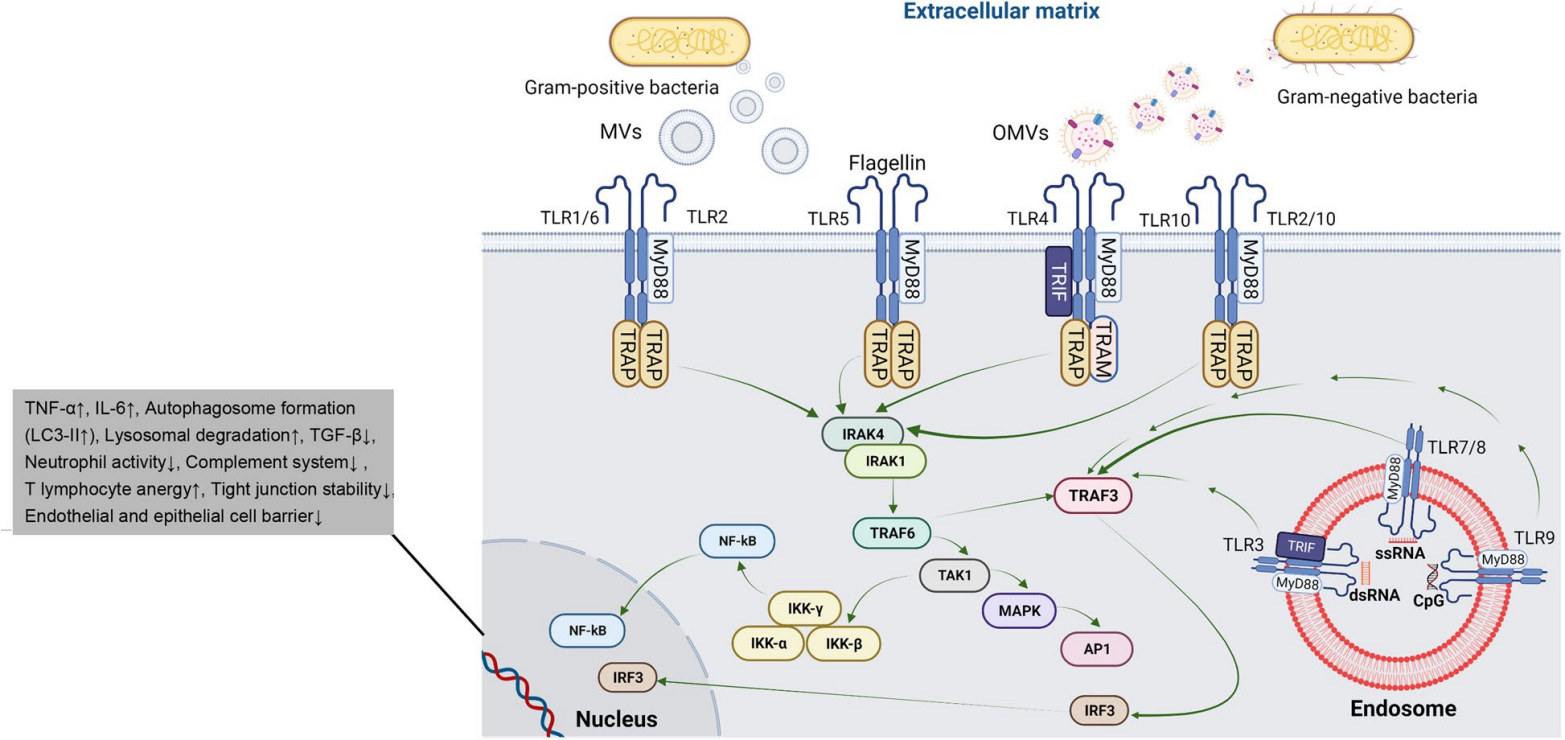
The possible effect of bacterial MVs and OMVs via engaging Toll-like receptor signaling pathway on different cell lineage

Of course, it should not be forgotten that OMVs can exert various cytopathic effects using different underlying mechanisms based on the parent bacteria. For example, *Acinetobacter baumannii* OMVs can increase the possibility of mitochondrial dysfunction in alveolar macrophages and pulmonary epithelial cells. The cytopathic properties of other bacterial EVs in the host gastrointestinal epithelium and mucosal barrier were indicated after exposure to OMVs of *Helicobacter pylori* [146], *Campylobacter jejuni* [147], *Treponema denticola* [148], and *Porphyromonas gingivalis* [149]. Histological examinations revealed the destruction of epithelial layers in gastrointestinal tissue and periodontium via affecting the function of tight junction proteins (i.e. zonula occludens and E-cadherin), and entry of oncogenic virulence factors such as cytotoxin-associated gene A. *Streptococcus pneumonia* MVs inhibit ECM neutrophil function and complement system activity. The exposure of *Staphylococcus aureus* MVs bearing alpha toxin can result in massive keratinocyte necrosis, eosinophilic reaction, and atopic dermatitis. Likewise, *Mycobacterium tuberculosis* MVs increase excessive CD4 lymphocyte activity and anergy [108, 150]. Group B *Streptococci* spp. produce MVs with the pontifical to loosen the integrity of natural cell barriers such as the blood-brain-barrier interface. Under such conditions, the recruitment of leukocytes, macrophages, and collagen degradation is increased [151]. Bacterial MVs can act as a two-edged sword in terms of infections inside the body. Alvarez-Jiménez et al. indicated that infection of human neutrophils with *Mycobacterium tuberculosis* (strain Mtb H37Rv) in in vitro conditions leads to the release of EVs which per se increases the intracellular elimination bacterial via macrophages activation, ROS production, and autophagic response. Mtb-bearing EVs can activate Toll-like receptors (TLR2 and 6) and co-stimulatory factors CD80 and CD86, with simultaneous induction of LC3-II, TNF-α, and IL-6, and reduction of TGF-β in macrophages compared to the groups exposed to natural bacteria-free EVs (Fig. 6) [9]. In another study, it was indicated that *Mycobacterium smegmatis* and *Mycobacterium avium*) infected macrophages can release EVs with potent inflammatory capacities. The higher levels of heat shock protein 70 on the surface of bacterial-infected EVs lead to the activation of TNF-α and NF-κB signaling pathways. These features coincide with active lysosomal activity, autophagolysosome formation, and elimination of intracellular bacteria [152].

### Interactions with bacteria

The reciprocal interaction between homogenous and heterogeneous bacteria via MVs has been reported [8, 52]. For instance, the fusion of *Lactobacillus acidophilus* MVs with other bacteria species, such as *Escherichia coli* and *Lactobacillus delbrueckii*. Upon the entry of *Lactobacillus acidophilus* MVs, the growth of bacteria is inhibited due to the delivery of an antimicrobial peptide bacteriocin [153]. Of note, the fusion of *Bacillus subtilis* lipophilic probe R18-labelled MVs with other *Bacillus subtilis* cells has been indicated [154]. The existence of degrading enzymes can facilitate the uptake of these particles by other bacteria [103]. Regarding the fact that MVs contain several quorum-sensing molecules such as N-acyl-homoserine lactones and Pseudomonas quinolone signal, it is logical to hypothesize that bacterial MVs are involved in interbacterial paracrine activity [1, 98, 155]. Factors like the blaOXA-24 gene that exists inside the MVs can induce resistance to carbapenem antibiotics in *Acinetobacter baumannii* cells [156]. Since EVs can fuse with bacterial membranes, likely, horizontal transfer of genes through EVs into bacteria is also possible [153, 154]. A similar process can occur in EV-embedded RNAs, in which recipient bacteria can utilize EVs-delivered RNAs [1].

## Purification of eukaryotic and prokaryotic MVs

In eukaryotic EVs, differentiation of each subpopulation was performed based on diameter size and the existence of specific surface protein markers such as tetraspanins (CD9, CD63, and CD81), heat shock proteins (Hsp70, Hsp90), MHC molecules and proteins involved in the biogenesis of MVBs (i.e. TSG101 and ALIX), and other factors like GTPases, SNAREs, Annexins, flotillins). Besides common factors, EVs harbor certain markers of their parent cells. Despite these features, the purification of EV subpopulations is difficult due to the overlap between some of these factors [6, 157]. Even though, poor purification efficiency can affect the clinical applications of EVs with varied therapeutic outcomes. The lack of a suitable purification method can lead to low-rate EVs and a highly heterogeneous EV population. To date, various isolation techniques have been introduced to isolate the EVs while preserving their therapeutic properties [6, 158–164]. Ultracentrifugation is the most routine approach in EV extraction. It was suggested that the formation of EV aggregates, and loss of functionality at higher speeds are the main disadvantages [143, 165]. Other methods such as filtration, precipitation, microfluidics, size exclusion chromatography, and affinity-based approaches are also used in EV isolation.

Due to efficient production and reduced toxicity of LPS, the application of detergents has been commercialized for OMV purification [166]. The characterization of bacterial MVs is done based on their shape, size distribution, concentration, surface or internal contents using electron microscopy, light scattering-based methods (e.g., nanoparticle tracking analysis and dynamic light scattering), and western blotting and/or flow cytometry [108]. Because none of these approaches can yield comprehensive data about MVs, a panel of these approaches is usually used [108]. It should not be forgotten that the bacterial source, culture conditions, and various parameters can affect the physicochemical properties of bacterial OMVs. For instance, in experiments conducted by Adriani et al. OMVs were isolated from bacteria grown in two different temperatures of 37 and 42 °C. Data confirmed that temperature can affect OMV protein content. Ultrastructural analysis has revealed a spherical electron-dense shape in both groups while the heterogeneity of isolated OMVs was high in groups exposed to the higher temperature. Likewise, the type of isolation can affect the structure of isolated OMVs. In the presence of deoxycholate detergent, the content of bacterial LPS is reduced in OMVs while the immunogenicity rate is not affected [166]. Recently, density gradient centrifugation has been performed as a common and most recommended method for MVs purification from samples. This approach is eligible to eliminate other cellular structures such as flagella and protein aggregates. Like eukaryotic EVs, size exclusion chromatography is also applicable for the isolation of bacterial MVs [27]. Despite both density gradient centrifugation, size exclusion chromatography can be used for the isolation of prokaryotic MVs, density gradient centrifugation is a time-consuming and labor-intensive method for MVs isolation and is not suitable for high-throughput applications while size exclusion chromatography is less time-consuming and can separate a generic mixed population of MVs [27, 167, 168].

Singorenko et al. claimed that a single purification method could be used to isolate the bacterial MVs from every strain within a bacteria species. Notably, data indicated that there is little difference in molecular patterns of OMVs isolated from UPEC536 and Nissle 1917 strains of *Escherichia coli* using density gradient centrifugation and size exclusion chromatography methods. Interestingly, *Escherichia coli* strain UPEC536 OMVs exhibited more homogeneity in size, density, and molecular contents relatively. On the contrary, *Mycobacterium smegmatis* MVs are heterogeneous and more problematic for the application of simple size exclusion isolation [27]. In an infection environment with stress such as lack of iron, certain bacteria types such as *Mycobacterium tuberculosis*, *Escherichia coli*, and *Haemophilus influenza* can release MVs with different cargo and size [27, 169]. *Acinetobacter baumannii* strain ATCC19606 can produce various sizes of OMVs at the growth phase. To be specific, the size of OMVs is small in the early phase of the growth cycle while the size of OMVs becomes medium, or large in early-mid, and stationary log phases, respectively [170]. These data show the need to use optimized fractionation protocols for the isolation of MVs depending on bacterial strain and growth phase [27] (Table 3).

**Table 3:** Effects of prokaryotic EVs on eukaryotic cells

Bacterial EVs	Recipient cells	Contents	Functions	References
*Acinetobacter baumannii*	Human macrophages cells	Virulence factor: Omp33–36 porin	Apoptosis↑, and autophagy modulation	[134]
*Clostridioides difficile*	Human colorectal epithelial Caco-2 cells	A total of 262 proteins	Pro-inflammatory response↑ and cytotoxicity of colonic epithelial cells↑	[118]
*Vibrio cholerae*	Human intestinal cell lines	Outer membrane porins, i.e., OmpU and OmpT bioactive cholera toxin		[51]
*Helicobacter pylori*	Macrophage RAW264.7 cells	Epimerase_2 domain-containing protein (Epi_2D), Probable malate: quinone oxidoreductase (Pro_mqo), and Probable cytosol aminopeptidase (Pro_ca)	Th2 immune response↑	[171]
*Escherichia coli* Nissle 1917 and Commensal ECOR63	Intestinal Epithelial Cells	The tcpC gene, such as ECOR63.	ZO-1↑ and claudin-14↑, and claudin-2↓, and protects epithelial barrier function	[172]
*Salmonella typhimurium*	Human colorectal carcinoma (HTC116), breast cancer (MCF-7), and hepatocellular carcinoma (HepG2) cell lines		Anti-neoplastic activity↑, tumor volume↓, tumor growth (Ki-67↓), Caspase-3↑, Bax↑, Beclin-1↑, and CD49b↑, down-regulated the Angiogenesis (VEGF↓)	[140]

## OMVs/MVs as novel therapeutics

As above-mentioned, several studies have confirmed the existence of diverse biological roles for MVs/OMVs. Bacterial EVs are actively involved in gene transfer, biofilm formation, nutrient acquisition, pathogenesis, and defense promotion because of toxins and immunomodulatory products [microbe-associated molecular patterns (MAMPs)] [26]. Like eukaryotic EVs, emerging data have pointed to the fact that MVs are potential therapeutic targets and can be used as biological shuttles in the transfer of target compounds. Either eukaryotic EVs or bacterial OMVs/MVs can harbor biomaterials via circulation with suitable stability and on-target delivery efficiency (Table 4). To yield higher therapeutic outcomes, bacterial OMVs/MVs can be sophistically engineered [143]. Alves and co-workers used engineered *Escherichia coli* bacteria for the production of phosphotriesterase (PTE) enzyme. Data indicated that sequestrated PTE inside the OMVs exhibits better enzymatic activity compared to free soluble forms. These features demonstrate that OMVs are valid bioshuttles for drug stability and delivery purposes [173]. To this end, the elucidation of mechanisms associated with cellular uptake seems mandatory. Besides, parameters such as administration route and parent bacterial source are critical issues in terms of bacterial MV/OMV bio-distribution [143, 174]. Recent decades have witnessed the advent of engineered OMV/MV, as tumoricidal agents, in cancer biology. Compared to conventional medications, the application of OMVs/MVs has superior on-target efficiencies [26]. For example, siRNA- and drug-loaded *Escherichia coli* OMVs were successfully used for targeting HER2, an EGF receptor, on the surface of tumor cells. Data confirmed that the size of the tumor mass decreased without prominent side effects. The treatment of hepatocellular carcinoma HepG2 cells with *Lactobacillus rhamnosus* MVs led to the regulation of tumor cells [139, 175]. Based on data from different studies, antibiotic-loaded OMVs can be used for the control of bacteria in the target tissues. For example, Huang and co-workers reported the reduction of intestinal bacterial load in a mouse model after administration of antibiotic-loaded OMVs [176]. Using certain bacterial EVs, it is possible to cross the epithelial and mucosal barrier [177].

**Table 4:** Different approaches used in bacterial OMVs for different therapeutic purposes

Bacterial Species	Cargo Loaded	Loading Method	Application	References
*Klebsiella pneumoniae*	Doxorubicin-loaded OMVs	Incubation	Anti-tumor efficacy in non-small-cell lung cancer	[178]
*Escherichia coli*	Loading indocyanine green to modified OMVs with a synthesized αvβ3 integrin targeting ligand and arginyl-glycyl-aspartic acid	The fusion effect and electrostatic interaction	Melanoma	[179]
*Escherichia coli*	Spy ligation to factor hemoglobin protease on the surface of OMVs	Incubation	Vaccine design	[180]
*Escherichia coli*	Melanin	Genetic engineering of parent bacteria	Cancer	[181]
*Escherichia coli*	siRNA	Electroporation	Cancer therapy	[182]
*Escherichia coli*	NanoLuc Luciferase enzyme	Genetic engineering	Bioluminescence Imaging	[183]
*Escherichia coli*	Inhibitor of Indoleamine2,3-dioxygenase	Electroporation	Cancer Immunotherapy	[184]
*Escherichia coli*	NanoLuc Luciferase enzyme	Genetic engineering	As modular nanodevices for biosensing and bioimaging	[185]
*Klebsiella pneumonia*	Doxorubicin	Incubation	Anti-tumor in non-small-cell lung cancer	[178]
*Pseudomonas aeruginosa*	Gold NPs	Electroporation	Drug delivery	[186]
*Escherichia coli*	PD-1 Plasmid	Engineered to express the targeted polypeptide LyP1	Cancer Immunotherapy	[187]
*Escherichia coli*	Mesoporous silica nanoparticles loaded with 5-FU	Ultracentrifugation	Drug delivery system	[188]
*Acinetobacter aumannii*	Antibiotics	ND	Vesicle-based drug efflux mechanism	[176]
*Salmonella typhimurium*	*Ovalbumin*	Genetic manipulation	Maturation of human monocyte-derived dendritic cells	[189]
*Escherichia coli*	Shiga toxin	Ultracentrifugation	Cytotoxicity assays	[190]

The global application of antibiotics has led to the emergence of resistant bacterial species and thus biologists and clinicians are looking for different modalities to overcome drug-resistant microbes. Prokaryotic EVs are de novo vaccine candidates for the prevention of different bacterial infections [26]. Both OMVs and MVs exhibit inherent immunogenicity for the stimulation of immune cells against bacteria. Because of relatively simple and cost-effective production and ease of application of almost all kinds of modern technologies for modification of bacterial EV contents and surface, these natural nanoparticles are future vaccination agents. It is postulated that several antigens can be simultaneously loaded into the EVs from parent bacteria especially non-replicating microbes. By refining the bacterial EV contents, it is possible to make them more resistant to degrading enzymes to strengthen their immunogenic and on-target efficiencies. In support of this notion, previous data have indicated that the treatment of OMVs with vaccination protocols and formulation did not alter their stabilities over time [26, 173]. In an experiment conducted by Huang et al., immunogenic properties of *Acinetobacter baumannii* OMVs (AbOMVs) were examined in in vitro and in vivo settings. The injection of AbOMVs led to the production of specific IgG in a mouse model. The combination of anti-serum with quinolone antibiotics yielded proper bactericidal effects. Data indicated that simultaneous administration of levofloxacin with anti-AbOMVs anti-serum reduces the bacterial load in pulmonary tissues and spleen. The levels of recruited immune cells are also decreased within the lung parenchyma [191]. Kim and co-workers proved that *Escherichia coli* OMVs can stimulate the immune system response and thus reduce the lethal rate in infected mice via the production of certain cytokines such as IFN-γ and IL-17 [192]. Other experiments have shown the immunogenic properties of *Neisseria meningitidis*, *Vibrio cholera*, *Salmonella typhimurium*, and *Staphylococcus aureus* OMVs/MVs as novel vaccination tools [26, 191, 193, 194]. Furthermore, many studies have shown the potential of EVs derived from Gram-negative and Gram-positive bacteria in vaccine evolution. Bacterial EVs are being expanded as vaccines against some bacterial infections induced by *Klebsiella pneumoniae, Bordetella pertussis*, *Vibrio cholerae*, *Clostridium perfringens*, *Salmonella typhimurium*, *Streptococcus pneumoniae,* and *Staphylococcus aureus*. Previous data have indicated the stimulation of cellular and humoral immune responses in vaccinated animals with bacterial EVs, leading to the reduction of bacterial load and infection. Indeed, EV-based vaccines have immune-stimulatory efficacy comparable to inactivated whole-cell vaccines [195, 196].

On the other hand, bacterial EVs can be used as effective and safe adjuvants for the development of certain vaccine types to elevate and regulate the immune response. For example, *Neisseria lactamica* EVs exhibited the powerful adjuvant activity against hepatitis B virus surface vaccine antigens [197]. Also, it has been reported that intranasal vaccination with *Escherichia coli* EVs can provoke immune system responses against the malaria without any side effects or weight loss in mice model, and antibody titers were comparable with other common adjuvants (e.g., MF59C.1 and cholera toxin) [198].

Despite these advantages, the application of BEVs should be done under special consideration. For instance, it was suggested that *Bacteroides fragilis* OMVs can stimulate the proliferation of cancer cells more than normal cells [199]. Besides, another side effect related to microbial MVs application is an excessive inflammatory response. Prolonged inspiration of BEVs promotes the recruitment of immune cells such as neutrophils and Th17 T lymphocytes into the pulmonary tissue, resulting in chronic pathological conditions and anaplastic changes [200]. The direct exposure of gastric epithelial cells to *Helicobacter pylori* OMVs can increase in situ levels of different cytokines such as IL-1β, − 6, and TNF-α by macrophages and IL-17 and INF-γ by T lymphocytes. Inflamed epithelial cells also release IL-8 because of virulent factors vacuolating cytotoxin A and cytotoxin-associated gene A [201]. Bacterial lipids, mainly LPS, are endotoxic and can facilitate bacterial virulence [202]. The mutual interaction between the bacterial via EVs can lead to changes in growth rate and invasion [203]. *Staphylococcus aureus* MVs can facilitate interbacterial communication, resulting in antibiotic resistance and changes in EV release. The prominent heterogeneity, significant variation between batches, and standard production protocols make the application of bacterial EVs problematic in in vivo conditions.

## Conclusions

Recent data have shown the similarity between eukaryotic EVs and prokaryotic MVs in terms of size, luminal contents, structure, etc. Compared to eukaryotic EVs, bacterial MVs possess relatively homogenous particle sizes [27]. Like Exos and microvesicles, bacterial MVs, and OMVs are alternates to synthetic polymeric micelles and liposomes in the delivery of therapeutics and specific signaling molecules to the targeted sites. Due to inherent immunogenic properties, both naïve and engineered bacterial MVs/OMVs can be used for vaccination and alleviation of pathological conditions. Because of specific features such as ease of production on large scales, it seems that these particles will be used as magic bullets for therapeutic purposes to reduce the possibility of antibiotic resistance and cancer patients.

## Data Availability

Not applicable.

## Abbreviations

AbOMVs: *Acinetobacter baumannii* OMVs
CMVs: Cytoplasmic membrane vesicles
Exos: Exosomes
EOMVs: Explosive outer-membrane vesicles
ECM: Extracellular matrix
EVs: Extracellular vesicles
ILVs: Intraluminal vesicles
LPS: Lipopolysaccharide
MVs: Membrane vesicles
MVBs: Multivesicular bodies
NLR: NOD-like receptor
NOD: Nucleotide-binding oligomerization domain
OMVs: Outer membrane vesicles
OM: Outer membrane
OIMVs: Outer-inner membrane vesicles
PAMPs: Pathogen-associated molecular patterns
PLs: Phosphoglycerolipids
PSMs: Phenol-soluble modulins
PTE: Phosphotriesterase
SyBVs: Synthetic bacterial vesicles
TLRs: Toll-like receptors
